# A Semi-Automated RGB-Based Method for Wildlife Crop Damage Detection Using QGIS-Integrated UAV Workflow

**DOI:** 10.3390/s25154734

**Published:** 2025-07-31

**Authors:** Sebastian Banaszek, Michał Szota

**Affiliations:** 1 Institute of Geodesy and Cartography, 27 Modzelewski Street, 02-679 Warsaw, Poland; 2 Fire Academy, 52/54 Slowackiego Street, 01-629 Warsaw, Poland; mszota@apoz.edu.pl

**Keywords:** UAV, crop damage, vegetation indices, RGB, QGIS

## Abstract

**Highlights:**

**What are the main findings?**

1. A semi-automated method was developed for detecting maize crop damage using UAV-acquired RGB imagery, fully integrated within the QGIS environment.
2. The method uses vegetation indices (ExG, GLI, MGRVI) and unsupervised k-means clustering, with interactive result tuning via a dedicated QGIS plugin.

**What is the implication of the main finding?**

1. The proposed approach enables fast, repeatable, and low-cost wildlife damage assessments without the need for multispectral sensors or artificial intelligence.
2. The method can be operationally used by non-specialists without GIS or coding skills, making it ideal for farmers, field technicians, and local environmental managers.

**Abstract:**

Monitoring crop damage caused by wildlife remains a significant challenge in agricultural management, particularly in the case of large-scale monocultures such as maize. The given study presents a semi-automated process for detecting wildlife-induced damage using RGB imagery acquired from unmanned aerial vehicles (UAVs). The method is designed for non-specialist users and is fully integrated within the QGIS platform. The proposed approach involves calculating three vegetation indices—Excess Green (ExG), Green Leaf Index (GLI), and Modified Green-Red Vegetation Index (MGRVI)—based on a standardized orthomosaic generated from RGB images collected via UAV. Subsequently, an unsupervised k-means clustering algorithm was applied to divide the field into five vegetation vigor classes. Within each class, 25% of the pixels with the lowest average index values were preliminarily classified as damaged. A dedicated QGIS plugin enables drone data analysts (Drone Data Analysts—DDAs) to adjust index thresholds, based on visual interpretation, interactively. The method was validated on a 50-hectare maize field, where 7 hectares of damage (15% of the area) were identified. The results indicate a high level of agreement between the automated and manual classifications, with an overall accuracy of 81%. The highest concentration of damage occurred in the “moderate” and “low” vigor zones. Final products included vigor classification maps, binary damage masks, and summary reports in HTML and DOCX formats with visualizations and statistical data. The results confirm the effectiveness and scalability of the proposed RGB-based procedure for crop damage assessment. The method offers a repeatable, cost-effective, and field-operable alternative to multispectral or AI-based approaches, making it suitable for integration with precision agriculture practices and wildlife population management.

## 1. Introduction

Crop damage caused by wildlife presents a significant challenge for agricultural production, particularly in monoculture systems, such as maize, which are highly attractive to large herbivores like wild boars and deer. In Central Europe, wild boars are among the primary sources of crop damage, with over 9800 reported cases over 20 years [1]. These incidents pose significant challenges due to seasonal feeding behavior and dispersed occurrence [2]. In many regions, the economic impact of such damage has intensified due to the increasing population of wild animals and fragmented monitoring systems [3,4,5]. Traditional damage assessment methods—based on field inspections and expert evaluations—are time consuming, offer low spatial precision, are difficult to scale, and often lack repeatability [3].

Remote sensing technologies, especially unmanned aerial vehicles (UAVs), have become effective tools for crop monitoring [4,6,7]. Their ability to capture high-resolution imagery allows for the detection of subtle spatial differences in plant condition. While many studies focus on the application of multispectral and hyperspectral sensors for vegetation assessment [8], there is still a practical gap in the operational use of UAV-based RGB data for damage inventory. RGB sensors are significantly cheaper and more accessible, yet their analytical potential remains underutilized in field applications.

Vegetation indices derived from RGB bands, such as Excess Green (ExG), Green Leaf Index (GLI), and Modified Green-Red Vegetation Index (MGRVI), show promising potential in detecting variations in plant vigor and early signs of stress [9,10,11]. However, most existing approaches rely on supervised machine learning models or datasets labeled by experts, which limits their applicability outside academic environments. Moreover, the lack of integration with user-friendly GIS tools hinders the scalability of such methods.

The article aims to present the Hybrid clustering with interactive threshold tuning (QGIS-integrated) method in detail and assess its effectiveness in the context of semi-automated detection of wildlife damage in agricultural crops. The outline of the paper is as follows. The study presents a semi-automated process for detecting crop damage caused by wildlife, based on UAV-derived RGB imagery, calculated vegetation indices, and a custom processing workflow integrated with QGIS. The method was designed with non-specialist users in mind—referred to as Drone Data Analysts (DDAs)—and operates within an open-source environment. Key elements of the solution include unsupervised clustering of vegetation vigor, adaptive thresholding for damage identification, and interactive calibration using Python-based QGIS tools. The research aims to evaluate the effectiveness, usability, and repeatability of the proposed approach in detecting wildlife-induced damage in maize fields. The method is based on the sixth generation of the data processing algorithm, with five preceding versions ultimately rejected. Its performance was assessed in terms of automation level, accuracy, and ease of integration with real-world agricultural monitoring procedures. During the study, several alternative crop damage detection methods using UAV data were tested, including proprietary approaches: Rule-based thresholding with expert-labeled raster reference, Heuristic index filtering with spatial grid aggregation (FITSH framework), Index-based spectral thresholding with resolution analysis, Iterative classification with multi-resolution sensitivity testing, and Weighted spectral scoring for binary classification (single-class model). However, the methods proved insufficient in terms of flexibility, result repeatability, and integration with open GIS environments. In response to the limitations, a hybrid approach based on unsupervised clustering and RGB index analysis with interactive threshold tuning was developed, fully integrated with QGIS and supported by custom Python scripts. The paper closes with discussion and conclusions.

## 2. Materials and Methods

### 2.1. Design, Evaluation, and Selection of Methods

Before implementing the final solution, a series of experimental approaches was developed and tested. They included five proprietary methods:

(1) Rule-based thresholding with expert-labeled raster reference—based on manually labeled reference maps and predefined decision thresholds. RGB imagery and expert-generated reference masks were used. The output consisted of binary damage masks used primarily for validation.
(2) Heuristic index filtering with spatial grid aggregation (FITSH framework)—relied on identifying value ranges of RGB indices within a spatial grid. Zonal analysis and heuristic filtering enabled the evaluation of damage within defined units. The result was an aggregated score per grid cell, useful in semi-automated field operations.
(3) Index-based spectral thresholding with resolution analysis—focused on sensitivity analysis of spectral thresholds at different spatial resolutions. RGB imagery with various GSD levels generated binary masks for benchmarking spectral variability.
(4) Iterative classification with multi-resolution sensitivity testing—involved an iterative classification process assessing result stability under changing raster resolution. RGB imagery and vegetation indices were used to generate multiclass classifications and assess spectral sensitivity.
(5) Weighted spectral scoring for binary classification (single-class model)—a scoring method based on RGB index thresholds and predefined weights (e.g., ExG, GLI, MGRVI). The result was a binary damage mask based on cumulative index weight scores.

All the approaches were rejected due to insufficient stability, low classification precision, or lack of integration with GIS systems. Most importantly, they were difficult to implement in field conditions and too complex for non-specialist drone data analysts (DDAs). Nonetheless, they provided essential insights during the iterative development of the final solution.

### 2.2. Comparative Evaluation of Developed Approaches

A systematic comparative evaluation of six alternative approaches was conducted before adopting the final solution based on Hybrid clustering with interactive threshold tuning (QGIS-integrated, the V6 method). Each method varied in analytical structure, degree of automation, and operational utility. A summary comparison of the individual approaches is presented in Table 1.

A multi-criteria decision analysis (MCDA) model was employed to support the evidence-based selection of the optimal method. The model incorporated six key criteria reflecting both technical performance and field applicability (Table 2).

The weights were defined based on expert consultation and iterative testing during the method development phase, reflecting operational needs and field applicability.

**Figure 1:**
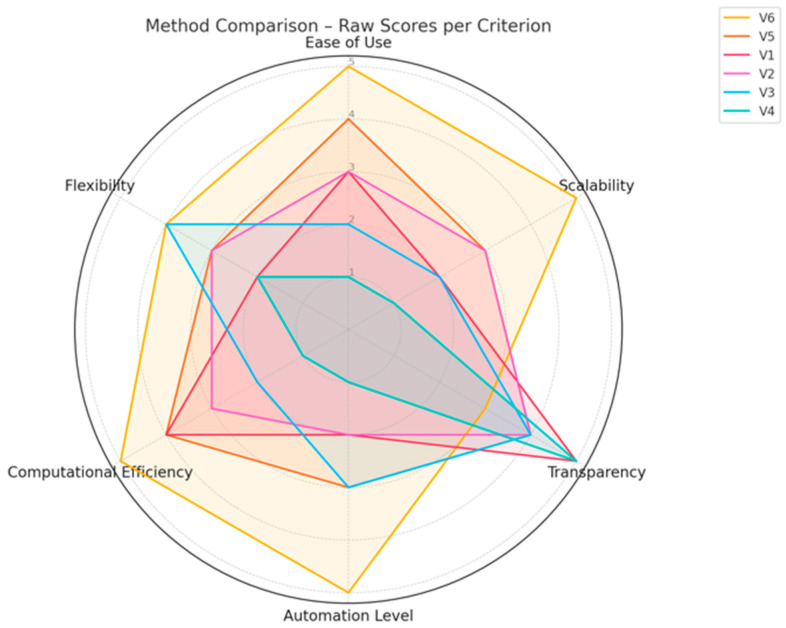
Method comparison—raw scores per criterion. Source: Authors’ own study, based on internal experimental trials.

Each method was scored on a scale from 0 to 1 across the six criteria, applying appropriate weighting factors. The final ranking is presented in Table 3 and visualized in Figure 1.

The most important technical challenges and decisions made during the development and selection process included:

(1) Exclusion of the VARI index—due to its unstable distribution and low classification value.
(2) Balancing automation and transparency—achieved through a semi-automated thresholding mechanism involving DDA operator input.
(3) Streamlining BAT file structure—integrating three Python scripts into a single process (DD_02.bat), eliminating external dependencies, and simplifying logic for DDA users.
(4) Iterative GUI tuning—refining the interface for manual threshold calibration in DD_03.bat.
(5) Final data integration—restructuring DD_04.bat to enable mask merging and generation of two report types: technical (HTML) and interpretive (DOCX).

The V6 method was selected as the final solution due to its optimal usability, scalability, and automation balance. Its integration with QGIS and exclusive reliance on RGB data ensured high repeatability, a low entry barrier for end users, and adaptability without the need for advanced scripting or calibration data. However, the use of unsupervised clustering presents some interpretive challenges; overall performance and operational value led to the method being recognized as the most suitable for field-based detection of wildlife-induced crop damage.

Six independent approaches to damage detection using UAV data were developed and evaluated in the research phase. Five of them (V1–V5) were ultimately rejected after testing. The final approach, the V6 method, was selected based on its efficiency, usability, and suitability for field conditions.

The V6 method approach was chosen due to:

(1) Full integration with QGIS.
(2) Processing entirely based on RGB data (no need for multispectral inputs).
(3) High user-friendliness (suitable for non-programmers and those without GIS expertise).
(4) Adjustable thresholds per class through GUI adaptation to field variability.
(5) Balanced repeatability, processing speed, and scalability.

The adopted methodology proved ready for operational field use and ideal for semi-automated detection of wildlife damage in various crops and environmental conditions.

### 2.3. The V6 Method—Hybrid Clustering with Interactive Threshold Tuning (QGIS-Integrated)

The V6 method approach was designed with the practical needs of field analysts (Drone Data Analysts—DDAs) in mind, ensuring a balance between analytical precision, ease of use, and result repeatability. The following design objectives were adopted:

(1) Operational simplicity—all stages can be executed using batch files and graphical tools in QGIS, without requiring knowledge of Python or advanced GIS functionalities.
(2) Speed—full analysis and reporting can be completed within a single working day.
(3) Low cost—no need for multispectral sensors; standard UAV-derived RGB images are sufficient.
(4) Accuracy—damage estimation is based on statistical outliers within vegetation index classes.
(5) Repeatability—an automated processing workflow ensures consistent results.
(6) Flexibility—threshold values can be adjusted individually per class, allowing adaptation to different crops and seasons.
(7) Low entry threshold—RGB sensor use and open-source software make the method accessible to non-specialists.

The implementation approach of the V6 method was based on the following tools:

(1) GIS: QGIS 3.28 (Firenze).
(2) Python libraries: rasterio, numpy, scikit-learn, jinja2, matplotlib.
(3) Vegetation indices: Excess Green (ExG), Green Leaf Index (GLI), Modified Green-Red Vegetation Index (MGRVI).

**Figure 2:**
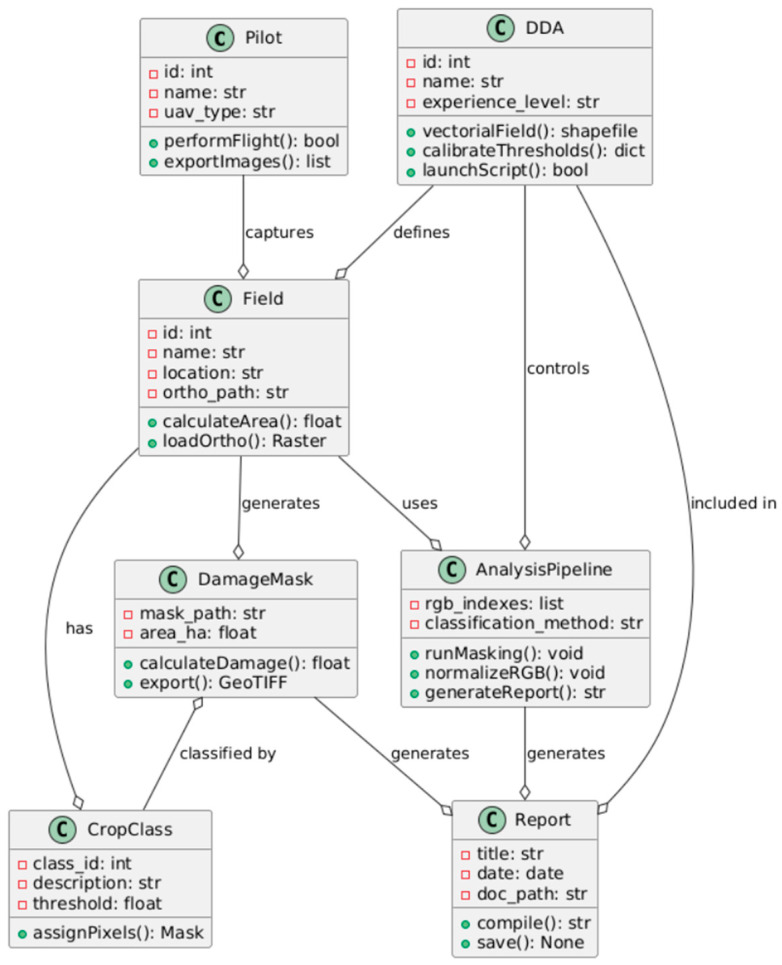
Extended UML Diagram for Drone-Based Field Assessment Pipeline. Source: Authors’ own study, based on internal experimental trials.

The data processing procedure within the V6 method is presented in Table 4 and illustrated in Figure 2.

For the implementation of the V6 method approach, four batch files (.bat) were developed, containing scripts that automate successive stages of the data processing workflow. A detailed description of the functionality of each file is provided below.

#### 2.3.1. DD_01.bat—QGIS Environment Initialization

Function: The DD_01.bat file is used to automatically create and launch a QGIS project with preconfigured paths, spatial reference system (EPSG:2180), an empty .qgz project file, and a set of auxiliary layers. The script:

(1) Detects the QGIS installation path.
(2) Creates the working directory structure.
(3) Initializes the QGIS project, enabling the DDA operator to begin work in a standardized environment.

Purpose: Standardizing the analytical environment allows for full reproducibility of the process regardless of the computer or user. It is crucial for interoperability and alignment with reproducible research principles.

#### 2.3.2. DD_02.bat—Raster Data Preprocessing

Function: The DD_02.bat file runs an integrated pipeline that includes:

(1) Clipping the orthomosaic based on the area.shp vector layer—eliminating pixels outside the cultivated area.
(2) Normalizing RGB channels—rescaling pixel values to the [0, 1] range.
(3) Calculating vegetation indices—ExG, GLI, and MGRVI.
(4) Crop condition classification—into five qualitative classes using the k-means algorithm.

Purpose: To convert raw imagery into biophysical indices and classify them, forming the basis for further damage detection. This step ensures an objective, algorithmic division of the agricultural space into homogeneous analytical units.

**Figure 3:**
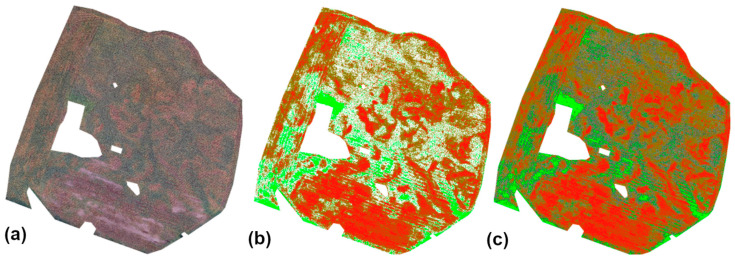
Ortho-image with five-class vigor map: (**a**) Ortho-image; (**b**) Vigor classification map; (**c**) Ortho-image + vigor classification map overlay. Color interpretation for (**b**,**c**): Bright green—very good, dark green—good, yellow—moderate, orange—poor, red—very poor. Colors correspond to vegetation vigor classes, as explained in the caption. Source: Authors’ own study, based on internal experimental trials.

Figure 3 shows the result of executing DD_02.bat: the orthomosaic clipped to the crop mask, vegetation masks generated for the clipped orthomosaic, and all masks overlaid on the orthomosaic.

#### 2.3.3. DD_03.bat—Interactive Detection of Wildlife Damage

Function: The DD_03.bat file allows graphical execution of the damage classification script within the QGIS Processing Toolbox. The script allows the user to:

(1) Specify four raster layers: crop condition classes, ExG, GLI, and MGRVI.
(2) Set threshold filtering parameters (default: 25% weakest pixels).
(3) Run the damage detection algorithm for individual classes using custom parameters, generating a damage mask for each crop class.

Purpose: To enable flexible control over classification parameters without modifying the source code, facilitating method adaptation by users without programming experience. Sequential processing of multiple classes allows iterative refinement of the results.

**Figure 4:**
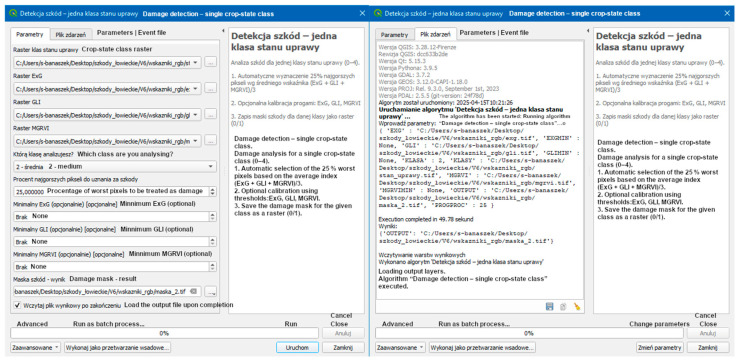
GUI interface for manipulating mask-generation indicators (tab: Parameters, Event file). Source: Authors’ own study, based on internal experimental trials.

Figure 4 shows the GUI of the DD_03.bat script in the QGIS environment using the Processing Toolbox.

**Figure 5:**
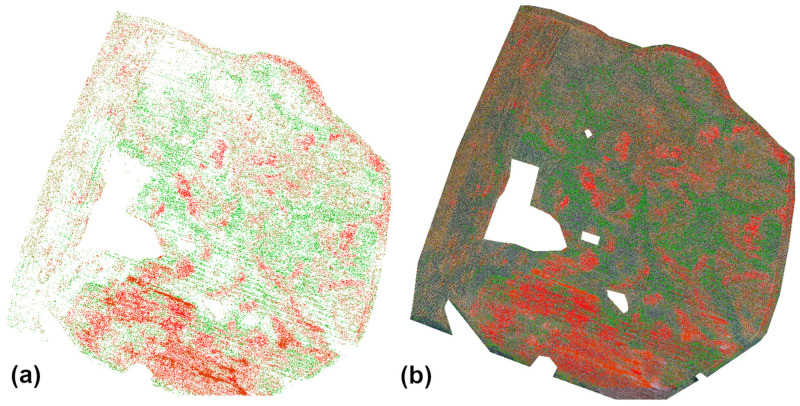
Damage detection masks generated for each crop vigor class and combined result: (**a**) Aggregated damage mask with color-coded vegetation vigor classes; (**b**) Binary mask of all detected damage overlaid on the RGB orthophoto. Color interpretation for (**a**): Bright green—very good, dark green—good, yellow—moderate, orange—poor, red—very poor. Each color represents damaged areas identified within the corresponding vegetation vigor class. Source: Authors’ own study, based on internal experimental trials.

Figure 5 presents the results of running DD_03.bat: individual masks generated for each vegetation vigor class, all masks combined, and masks overlaid on the orthomosaic.

#### 2.3.4. DD_04.bat—Generating the Interpretive Report (.docx)

Function: The DD_04.bat file generates both a technical report in HTML format and an interpretive report in Microsoft Word (.docx) format containing:

(1) Identification data (field, location, UAV flight date, institution).
(2) Crop and damage area statistics (total and by class).
(3) Tabulated summary of damage shared in each class.
(4) Graphical attachments (damage chart, damage masks, original orthomosaic).

Input data is interactively collected from the user (DDA) during script execution.

Purpose: Automating the generation of final documents increases process transparency, facilitates result archiving, and supports communication (e.g., between DDA, clients, farmers, authorities). The .docx format allows for further report editing and adaptation to formal requirements.

Nine major development iterations of the V6 method procedure were conducted, focusing on index selection, normalization methods, and crop condition class calibration. More than 30 units and integration tests were performed to verify computational stability, threshold optimization, graphical coherence, and visual consistency of results.

## 3. Results

**Figure 6:**
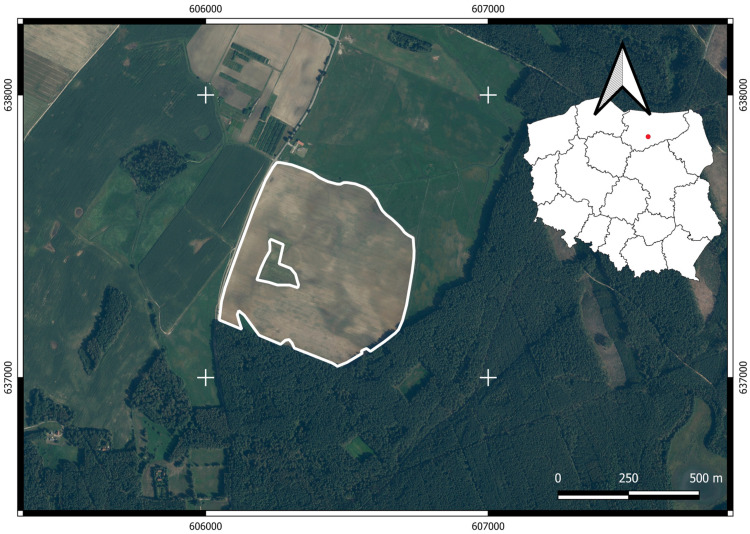
Location of the experimental area in northeastern Poland, including a detailed view of the field boundaries overlaid on high-resolution aerial imagery, and its general position within the national context. Source: Authors’ own study, based on internal experimental trials.

The developed procedure was tested on an orthomosaic generated from 222 RGB images captured by a UAV, covering a 0.66 km^2^ maize cultivation area with confirmed wildlife damage (wild boars). The UAV platform used in this study was a DJI Inspire One quadrotor equipped with the Zenmuse X3 RGB digital camera (featuring a 1/2.3” CMOS sensor, 12-megapixel resolution, 20 mm focal length). Flights were performed at a constant altitude of 150 m above ground level (AGL), with 75% forward overlap and 60% side overlap, resulting in a ground sampling distance (GSD) of 6,5 cm. The mission consisted of five separate flight sessions with a total duration of approximately 60 min. The research was carried out in the village of Baldy, located in northeastern Poland, in the Warmian–Masurian Voivodeship (approx. 53°38′ N, 20°42′ E). Figure 6 presents the location of the experimental area.

Data processing and analysis were conducted using open-source software: QGIS 3.28 LTR, OpenDroneMap/WebODM 2.7.1, and Python libraries 3.12 (OpenCV, NumPy, and scikit-image) using PC: OS Windows 10 Pro 64-bit, processor Intel Core i7-7700K CPU 4.20 GHz, 16 GB RAM, Intel Corporation, Santa Clara, CA, USA NVIDIA GeForce GTX 650, 8 GB, NVIDIA Corporation, Santa Clara, CA, USA.

The orthomosaic was generated using OpenDroneMap (2.7.1) and saved as a high-resolution raster file (.geotiff) in the EPSG:2180 coordinate reference system. The orthomosaic parameters are summarized in Table 5.

The entire test was performed in the QGIS 3.28 LTR environment, using Python 3.12 and the following libraries: rasterio, numpy, scikit-learn, matplotlib, python-docx, and pillow. All scripts and procedures were executed via dedicated batch files (.bat), which contained complete embedded logic and did not require external Python script calls.

During the test, the procedure was applied to a test field with an area of over 47 ha, where a total of 6.88 ha of damage was identified (14.6% of the area). Key parameters of the test execution are presented below:

(1) Area of cultivated field analyzed: 47.13 ha.
(2) Number of crop condition classes: 5.
(3) Total number of raster layers created by DDA: 17 (RGB orthomosaic, damage_class0.tif—damage_class4.tif, five damage masks for each crop class × three threshold correction levels).
(4) Total number of vector layers created by DDA: 1 (area.shp—crop mask vector).
(5) Automatically generated files from BAT scripts: minimum eight raster layers, graphical outputs, and reports.
(6) Number of threshold correction iterations: 3.
(7) Number of BAT file executions during testing and calibration: 4.
(8) Area of detected damage: 6.88 ha.
(9) Percentage of damaged area relative to total crop area: 14.6%.
(10) Total DDA working time (person-hours): <1 h.

The execution time and characteristics of each step are summarized in Table 6.

The test results include the following outputs:

(1) Crop vigor classification. An unsupervised classification of vegetation indices derived from RGB data using the k-means clustering method resulted in the generation of a five-class map reflecting different levels of plant vigor. The classes “very good,” “good,” “moderate,” “poor,” and “very poor” represent the relative health condition and canopy density of maize vegetation within the study area. Visual interpretation of the raster classification indicated a spatially coherent distribution of vigor zones. The central area of the field was dominated by “very good” and “good” classes, while the edges and corners were characterized by “poor” and “very poor” conditions. The “moderate” class was the most prevalent and often formed transition zones between healthy and weakened areas.
(2) Detection of wildlife damage. Within each vigor class, 25% of the pixels with the lowest average vegetation index values (mean of ExG, GLI, and MGRVI) were preliminarily marked as potentially damaged. This percentile-based thresholding allowed for the identification of localized zones with abnormally low vegetation index values, indicative of potential wildlife disturbance. The damage masks generated for each class were aggregated into a single binary raster representing the potentially affected area. Manual threshold adjustment for each class using the QGIS plugin enabled the results to be aligned with visual interpretation and field knowledge, improving agreement between algorithmic outputs and actual canopy disruptions.
(3) Quantitative assessment. The total area classified as damaged across all vigor classes amounted to 6.88 ha, representing 14.6% of the total crop area. Damage distribution was not uniform across vigor classes—the highest concentration of damage was found in the “moderate” and “poor” categories, accounting for 2.31 ha and 2.68 ha, respectively.

A detailed breakdown of the inventoried damaged areas by crop vigor class is provided in Table 7.

The largest share of detected damage was recorded in moderate and declining crop condition zones, suggesting that wildlife activity is more frequent in areas already exhibiting signs of weakening. In contrast, the “very good” class showed minimal disturbance, which aligns with the expected avoidance of dense vegetation by foraging animals.

### 3.1. Accuracy Assessment of Crop Condition Classification

In order to validate the reliability of the automatic crop condition classification derived from RGB-based vegetation indices, an accuracy assessment was conducted using a set of manually interpreted reference points. The classification followed a five-level scale, consistent with the automated output:

(1) 0—Very Good: uniform, dense canopy, vivid green.
(2) 1—Good: slight loss of vigor, small signs of thinning or stress.
(3) 2—Moderate: visible degradation, irregular canopy.
(4) 3—Poor: advanced degradation, yellowing or patchy areas.
(5) 4—Very Poor: severe degradation, almost no vegetation cover.

**Figure 7:**
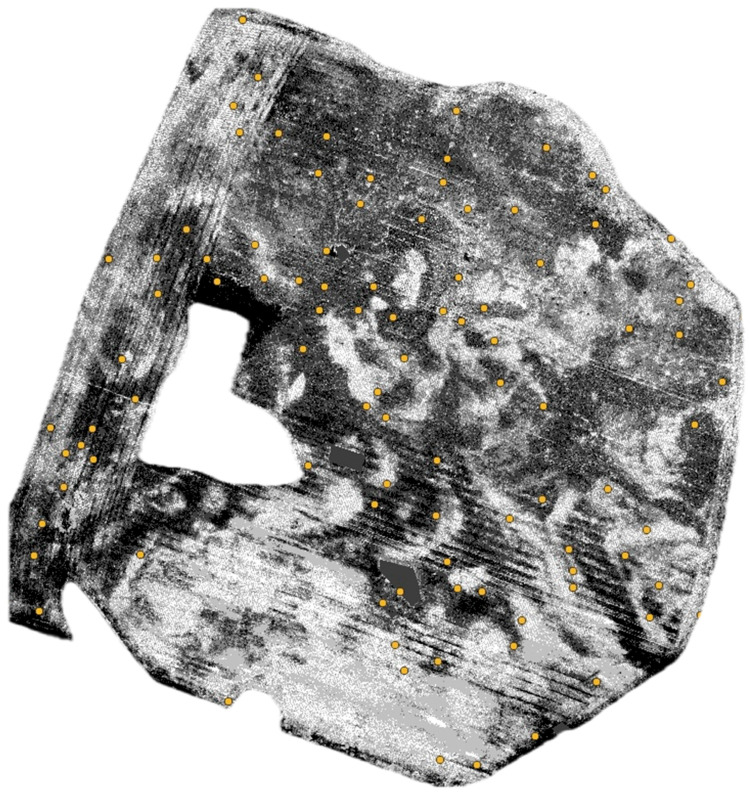
Location of control points randomly generated in QGIS. Source: Authors’ own study, based on internal experimental trials.

A total of 100 sample points were randomly distributed across the study field using QGIS, with a minimum spacing of 15 m and a fixed random seed (45). Figure 7 presents the location of control points randomly generated in QGIS.

Each point was visually assessed by an expert using the original UAV orthophoto and labeled according to the classification system. These labels served as ground truth values.

The automated classification results were then extracted for the same locations and compared against the manual reference. The evaluation focused on standard classification metrics: precision, recall, and F1-score for each class, alongside overall accuracy and macro-/weighted averages.

**Figure 8:**
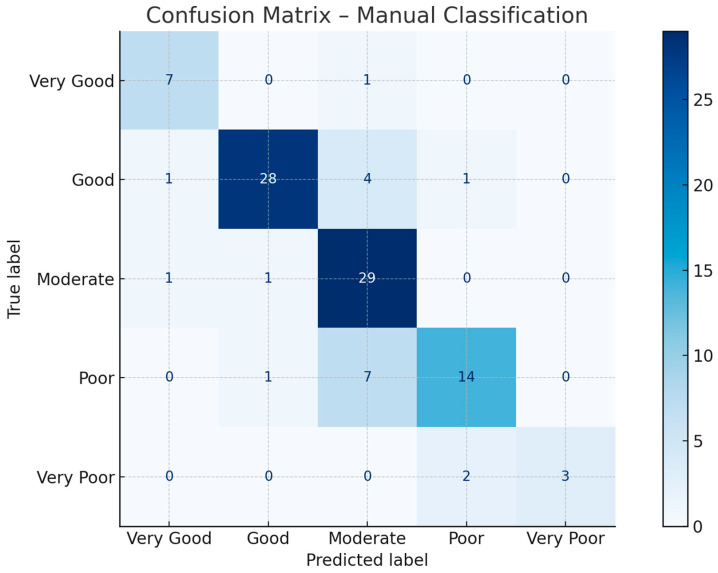
Confusion matrix for the conducted accuracy assess. Source: Authors’ own study, based on internal experimental trials.

The results indicate a high level of agreement between the automated and manual classifications, with an overall accuracy of 81%. Class-wise performance varied slightly, with the ‘Good’ and ‘Moderate’ categories showing the highest support and robust metric values. Discrepancies primarily stemmed from the transitional nature of crop conditions and the inherent subjectivity of visual interpretation. A summary of the classification metrics is presented in Table 8. The generated confusion matrix is presented in Figure 8.

These results confirm the operational validity of the proposed classification pipeline for field-scale applications, particularly for initial condition assessment prior to damage detection.

### 3.2. Summary of the Conducted Study

A complete, multi-stage procedure was developed and tested for inventorying wildlife-induced damage in maize crops using UAV-acquired RGB orthomosaics as part of the research and development work. The main objective was to create a method enabling fast, repeatable, and cost-effective data processing without requiring advanced knowledge of GIS, photogrammetry, or programming. To achieve this, a set of tools was prepared in the form of batch (.bat) files that enable automatic and semi-automatic execution of analytical modules and integrated interfaces.

The results indicate high effectiveness and repeatability of the algorithms, stable performance of the interfaces, and short data processing time. The procedure enables the generation of a complete analytical report within one working day on a standard-performance laptop (from the moment the orthomosaic is obtained), representing a significant improvement in the decision-making process for precision agriculture. The advantages of the V6 method are summarized in Table 9.

The developed procedure can be used operationally by users without advanced GIS expertise, serving as a decision-support tool in precision agriculture and damage compensation procedures related to wildlife. Iterative calibration of thresholds and classifiers confirmed the method’s effectiveness and its adaptability to different crop conditions.

## 4. Discussion

The primary objective of the study was to develop a Hybrid clustering with interactive threshold tuning (QGIS-integrated) method that enables fast, repeatable, and cost-effective data processing—accessible to non-specialist users without the need for advanced knowledge of GIS, photogrammetry, or programming. Accordingly, the following section of the discussion presents a comparative analysis of the proposed method against other existing approaches for crop damage assessment.

Compared to DSM-based and deep learning methods for maize damage detection [4], the V6 method balances interpretability, flexibility, and data economy. While the DSM method uses LiDAR-derived canopy height and the deep learning model uses QGIS-embedded neural networks, the V6 method integrates clustering and user-defined thresholds in RGB indices within QGIS. It avoids reliance on costly sensors and supports expert-guided classification, enhancing sensitivity without sacrificing specificity. These features make the V6 method a robust framework for field-level assessments where flexibility and transparency are critical.

The deep learning-based classification approach, employing architectures such as U-Net and SegFormer, relies on annotated datasets and high computational resources [7]. The V6 method, by contrast, offers a transparent, user-guided alternative through interactive thresholding. Fully integrated with QGIS, it is better suited for contexts with limited data availability or processing capacity.

The supervised SVM classification approach requires training datasets and manual extraction of zones [4]. The V6 method, through unsupervised clustering and rule-based scoring, eliminates the need for extensive training data, offering a more adaptable and interpretable solution, particularly in dynamic or under-resourced contexts.

Compared to the use of ensemble models for predicting wildlife conflict risk zones [5], the V6 method enables high-resolution mapping of actual damage based on spectral thresholds. The integration of both approaches has the potential to create a powerful, multi-scale framework for proactive conflict management.

The results obtained using our proposed V6 method demonstrate a robust and adaptable approach to detecting damage in agricultural plots using UAV-based RGB imagery. Alternatively, the approach based on the use of Sentinel-2 time series data combined with weather records enabled the detection and attribution of cereal yield losses at sub-field scales in South Australia [12]. While that method focused on phenological modeling of crop growth stages and thermal-time series to predict yield reductions using the Crop Damage Index (CDI), our method leverages interactive user-defined threshold tuning and clustering across multiple spectral indices (ExG, VARI, MGRVI), enabling high-resolution, explainable classification of fine-scale damage features such as wild boar rooting. Unlike the method that attributes yield losses to specific weather events, our approach aims to support rapid damage assessment under heterogeneous field conditions without relying on time-series or meteorological data. Nevertheless, both methods emphasize the importance of detailed spatial analysis and tailored indicators to enhance the reliability of operational crop damage assessment systems.

The method based on a Fully Connected Neural Network (FCNN) trained on hyperspectral indices for crop classification is highly accurate but requires extensive resources and advanced sensors [13]. The V6 method, in contrast, works with standard RGB UAV imagery, unsupervised clustering, and interactively adjustable thresholds in QGIS. Its simplicity and real-time adaptability make it particularly effective in non-specialist and rapid-response settings, offering a complementary alternative to high-precision deep learning systems. SepHRNet—an advanced crop-mapping architecture using deep learning with multi-head attention and ensemble learning—delivers high-resolution outputs but is computationally intensive and requires labeled data [14]. In contrast, the V6 method emphasizes accessibility and transparency in the crop damage assessment process. Although it is less semantically rich, it is well suited for rapid, local assessments under limited computational resources.

Research on the saturation of vegetation indices using 3D radiative transfer simulations has encountered theoretical limitations [15], whereas the V6 method applies a pragmatic selection of indices and a scoring system to overcome these challenges in dense vegetation conditions. Integrating insights from both approaches may further improve the application’s performance.

Some researchers have used DeepLabV3+ to assess rice field damage via Sentinel-2 imagery [16]. Their deep learning approach, although accurate, demands extensive preprocessing and GPU-based training. The V6 method offers a simpler, more accessible alternative requiring only RGB data and basic QGIS operations, making it suitable for immediate, field-level deployment and applications lacking comprehensive ground truth data.

In grapevine health assessments, where proximity sensors and multispectral AI-based methods dominate, the V6 method uses only RGB orthophotos, enabling high classification precision through expert-defined thresholds. Unlike deep learning models, the V6 method is transparent, adaptable, and suitable for rapid deployment in diverse agricultural contexts [17].

The use of satellite-based machine learning for monitoring sugarcane health is a scalable method, but it lacks interpretability [18]. The V6 method, through its integration with QGIS, offers immediate and interpretable results based on expert input—ideal for local decision-making and operational workflows.

In comparison to studies that used PlanetScope and SkySat data combined with ML and SAM models to detect greenhouse damage—where the pipeline is fully automated and satellite-based [19], the V6 method provides a transparent, flexible, and cost-effective UAV-based alternative, suitable for on-the-ground operations and environments with limited data access.

In the context of using long-term EVI/NDVI trends to monitor forest condition [20], the V6 method offers a short-term, high-resolution alternative based on UAV data and user-defined thresholds—ideal for immediate applications such as damage detection.

In the application of advanced sensor-based pest and disease detection methods, particular emphasis is placed on the integration of data acquired through multiple types of sensors [21]. The V6 method demonstrates that simpler systems using RGB data and interactive thresholds can also yield operationally useful results. QGIS integration allows for expert input and rapid calibration, offering a practical alternative in settings with limited access to sophisticated hardware.

While there are existing approaches that integrate remote sensing, artificial intelligence, and machine learning for monitoring and forecasting pests across large agricultural landscapes [22], our method focuses on more localized, high-resolution damage detection using RGB imagery acquired from UAVs. The key distinction lies in the scale and granularity of the application. The V6 method enables flexible and interpretable classification of crop damage based on tunable spectral indices thresholds, optimized interactively for specific field conditions. Unlike the large-scale ML models referenced by [22], which often require extensive training datasets and rely on generalized patterns, the V6 method supports semi-automated, user-adjustable workflows suitable for site-specific analysis and rapid implementation without high computational demands. It makes it particularly applicable for operational damage assessment and responsive agricultural decision-making in resource-limited settings. However, both approaches converge on the principle of enhancing sustainability and mitigating yield losses through geospatial intelligence and adaptive data processing.

The method developed by the authors presents a transparent, cost-effective solution for fine-scale crop damage assessment. Some authors proposed a decision support system focused on waterlogging detection using Sentinel-2 data and machine learning trained on parcel-level classifications and 20 vegetation indices [23]. Their system, while precise, requires cloud-free satellite imagery, annotated datasets, and supplementary layers (e.g., Corine). The method developed by the authors, however, operates without machine learning training, relying on unsupervised clustering and scoring rules. Its strengths lie in rapid deployability, compatibility with field data, and explainability, making it more suitable for time-sensitive, localized management. At the same time, the approach presented in [23] is better suited for broader-scale, automated analyses.

Flood damage mapping using SAR and optical satellite data within a multi-source system is effective for regional-scale analyses, and its performance depends on supervised classification and static thresholds [24]. The method presented in the article introduces adaptive, rule-based scoring with real-time feedback, enhancing precision under complex conditions.

YOLO-RS is another example of a state-of-the-art deep learning model optimized for object detection in remote sensing [25]. It achieves high accuracy but depends on large, annotated datasets and advanced configuration. The V6 method requires no training, instead offering unsupervised clustering and interactive thresholding in QGIS. It makes it particularly attractive for real-time field applications where speed, cost, and interpretability are critical.

In some studies, limitations in the selection of the vegetation index have been outlined [26]. The V6 method directly addresses this issue through user-guided index selection and threshold tuning within QGIS. The adaptability ensures accurate and transparent classification, improving upon rigid, fixed-index approaches.

Studies integrating socio-economic surveys and satellite data to investigate crop abandonment from wildlife damage provide valuable insights into long-term trends [27]. Our method enables real-time analysis based on high-resolution UAV digital imagery. This allows for drawing practical conclusions without the need for extensive datasets or long-term observations.

The method presented in the article is useful for policy implementation at the local level, offering tangible, spatial evidence for damage assessment using UAV imagery [28]. When combined with survey- and econometrics-based approaches related to agricultural land abandonment, it can provide essential validation for macro-level models.

## 5. Conclusions

The study presents a semi-automated, low-cost processing workflow for detecting crop damage caused by wildlife, based on RGB images acquired from UAVs and open-source GIS tools. By combining vegetation indices (ExG, GLI, MGRVI), unsupervised clustering, and interactive threshold tuning, the proposed method enables effective and repeatable damage assessment without the need for multispectral sensors or complex machine learning models.

The approach was tested on a 47.13-hectare maize field and successfully identified 6.88 hectares of damage (14.6% of the total area). The highest concentration of damage was observed in areas with “moderate” and “poor” crop vigor, consistent with the typical foraging behavior of wildlife. The integration of a QGIS-based plugin allowed non-specialist users (Drone Data Analysts—DDAs) to adjust classification thresholds and visually validate results, improving both accuracy and usability.

Compared to more complex or expensive methods, the presented solution offers a pragmatic alternative for operational crop monitoring and damage inventory. Its modular architecture, short processing time, and compatibility with standard RGB data make it well suited for implementation in precision agriculture and wildlife management practices.

Further development should focus on temporal extension (multi-date damage tracking), integration of structural features (e.g., canopy height models), and adaptation to other crop types and animal species. The results highlight the potential of accessible, affordable UAV-based RGB solutions to support spatially precise, data-driven decisions in modern agriculture.

## Figures and Tables

**Table 1:** Summary of Vegetation Damage Classification Methods on RGB Imagery.

Code	Method Name	Core Approach	Input Data	Output Type	Best Use Case
V1	Rule-based thresholding with expert-labeled raster reference	Fixed thresholds based on expert-labeled raster references	RGB + expert-defined masks	Binary damage mask	Validation against expert reference data
V2	Heuristic index filtering with spatial grid aggregation (FITSH framework)	Zonal analysis with heuristic filtering across a regular grid	RGB + fixed grid layer in GIS	Grid-wise aggregated damage scoring	Semi-automated deployment for field diagnostics and scouting
V3	Index-based spectral thresholding with resolution analysis	Threshold testing across multiple spatial resolutions	RGB imagery at varying GSD levels	Multi-resolution damage mask series	Resolution sensitivity benchmarking
V4	Iterative classification with multi-resolution sensitivity testing	Iterative reclassification and stability testing under resolution variance	RGB + computed vegetation indices	Sensitivity-aware multi-class damage classification	Algorithm robustness optimization and parameter tuning
V5	Weighted spectral scoring (binary single-class model)	Scoring model using weighted RGB indices with binary pass/fail rule	RGB + index thresholds (ExG, GLI, MGRVI)	Binary mask (damage/no-damage)	Controlled scoring-based experimental damage detection
V6	Hybrid clustering with interactive threshold tuning (QGIS-integrated)	K-means + quartile-based tuning + QGIS GUI	RGB only (ExG, GLI, MGRVI)	Interactive class-specific mask	Rapid analysis with in-field customization

Source: Authors’ own study, based on internal experimental trials.

**Table 2:** Evaluation Criteria for Vegetation Damage Detection Methods—Weights and Description.

Criterion	Symbol	Weight (%)	Description
Ease of Use	EOU	25	GUI-based usability and implementation simplicity
Scalability	SC	20	Applicability across different fields without retraining
Transparency	TR	15	Interpretability of logic and outputs
Automation Level	AUTO	15	Level of automation of the detection pipeline
Computational Efficiency	CE	15	Runtime and resource requirements
Flexibility	FLEX	10	Adaptability to crop types and conditions

Source: Authors’ own study, based on internal experimental trials.

**Table 3:** Ranking of Vegetation Damage Detection Methods by Weighted Overall Score.

Rank	Code	Method Name	Weighted Score
1	V6	Hybrid clustering-based index analysis with tuning (QGIS)	4.60
2	V5	Heuristic index filtering with FITSH framework	3.55
3	V1	Rule-based thresholding with expert raster	3.00
3	V2	Spectral thresholding with resolution analysis	3.00
5	V3	Iterative classification with sensitivity testing	2.65
6	V4	Weighted spectral scoring (single-class binary)	1.70

Source: Authors’ own study, based on internal experimental trials.

**Table 4:** Workflow Table—UAV-Based Damage Inventory Procedure, the V6 method.

Step No.	Step Description	Executor	Type of Operation	Tool
1	Flight execution	Pilot	AUTO	GCS
2	Generation of orthophotomap	DDA	AUTO	ODM/Pix4D/Agisoft
3	Preparation of data in the working directory	DDA	MANUAL	Windows Explorer
4	Creation of QGIS project and area.shp layer	BAT	AUTO	DD_01.bat
5	Vectorization of crop boundaries in QGIS (area.shp layer)	DDA	MANUAL	QGIS
6	Masking orthophoto, RGB normalization, index calculation, and crop state classification	BAT	AUTO	DD_02.bat
7	Damage detection for a single crop state class	DDA	SEMI	DD_03.bat
8	Repeat detection for all classes	DDA	SEMI	DD_03.bat
9	Mask integration, previews, and final report generation	BAT	SEMI	DD_04.bat
10	Archiving final data	DDA	MANUAL	Windows/QGIS
11	Preparation of technical note	DDA	MANUAL	Any editor

Source: Authors’ own study, based on internal experimental trials.

**Table 5:** Summary of parameters from the orthophotomap generation process in the ODM environment (unsupervised process).

Parameter No.	Parameter Name	Parameter Value
Processing Summary		
1	Reconstructed Images	222 over 222 shots (100.0%)
2	Reconstructed Points (Sparse)	98,584 over 440,539 points (22.4%)
3	Reconstructed Points (Dense)	19,070,369 points
4	Detected Features	6641 features
5	Reconstructed Features	2162 features
Reconstruction Details		
6	Average Reprojection Error (normalized/pixels/angular)	0.11/0.75/0.00028
7	Average Track Length	5.29 images
8	Average Track Length (>2)	5.33 images

Source: Authors’ own study, based on internal experimental trials.

**Table 6:** Evaluation of Scripted Geospatial Tasks in a Semi-Automated QGIS Environment (DD_01–DD_04).

Script	Description	Average Execution Time	Operational Effectiveness	Number of Tests	Lines of Code	Additional Data
DD_01.bat	Creating a QGIS project and the ‘area.shp’ layer	<1 min.	Automatic QGIS path detection: 94%	6	98	2 helper functions, fallback mechanisms: yes
DD_02.bat	Orthophoto processing and crop classification	<10 min.	Input file detection: 100%	12	267	4 raster layers, 4 PNGs, 6 Python libraries, nine raster operations, six intermediate files
DD_03.bat	Damage detection in crop condition classes	<30 min.	GUI launch success rate: 100%	10	181	5 classes, five masks, 8 GUI input fields, threshold adjustment range [0.0–1.0]
DD_04.bat	Mask integration and reporting	<5 min.	User input recognition: 100%	6	223	5 output files, three embedded graphics, automatic formatting (HTML + DOCX)

Source: Authors’ own study, based on internal experimental trials.

**Table 7:** Area and percentage of detected damage by crop vigor class.

Crop Vigor Class	Damage Area (ha)	Damage Share (%)
Very Good	0.13	0.3
Good	0.41	0.9
Moderate	2.31	4.9
Poor	2.68	5.7
Very Poor	1.35	2.8
Total	6.88	14.6

Source: Authors’ own study, based on internal experimental trials.

**Table 8:** Summary of the classification metrics.

Script	Description	Average Execution Time	Operational Effectiveness	Number of Tests
Very Good	0.778	0.875	0.824	8
Good	0.933	0.824	0.875	34
Moderate	0.707	0.935	0.806	31
Poor	0.824	0.636	0.718	22
Very Poor	1.000	0.600	0.750	5
Overall Accuracy			0.81	

Source: Authors’ own study, based on internal experimental trials.

**Table 9:** Overview of the V6 method Image Classification Capabilities and Requirements.

Feature	The V6 Method Capability
Sensor requirement	Only RGB
Software environment	Fully open source (QGIS + Python)
User skill requirement	Low (DDA-level; no coding)
Adaptability	Customizable thresholds per class
Automation level	High (auto classification + manual tuning option)
Result format	Spatial layers + docx report

Source: Authors’ own study, based on internal experimental trials.

## Data Availability

The data supporting the findings of this study are available on request from the corresponding author.

## Abbreviations

AI	Artificial Intelligence
AUTO	Automation Level
BAT	Batch file
CE	Computational Efficiency
DDA	Drone Data Analyst
DOCX	Microsoft Word Document
EOU	Ease of Use
EPSG	European Petroleum Survey Group
ExG	Excess Green
FLEX	Flexibility
GLI	Green Leaf Index
GSD	Ground Sampling Distance
GUI	Graphical User Interface
HTML	HyperText Markup Language
MCDA	Multi-Criteria Decision Analysis
MGRVI	Modified Green-Red Vegetation Index
ML	Machine Learning
ODM	OpenDroneMap
QGIS	Quantum Geographic Information System
RGB	Red-Green-Blue
RS	Remote Sensing
SC	Scalability
TR	Transparency
UAV	Unmanned Aerial Vehicle
UML	Unified Modeling
